# A novel force scaling method for ground reaction force estimation from pressure insoles to enable long-term remote monitoring

**DOI:** 10.1371/journal.pone.0358957

**Published:** 2026-10-26

**Authors:** Karl E. Zelik, Katherine M. Rodzak, Meredith K. Owen, Brian Noehren

**Affiliations:** 1 Department of Mechanical Engineering, Vanderbilt University, Nashville, Tennessee, United States of America; 2 Department of Biomedical Engineering, Vanderbilt University, Nashville, Tennessee, United States of America; 3 Department of Physical Medicine and Rehabilitation, Vanderbilt University, Nashville, Tennessee, United States of America; 4 Department of Physical Therapy, University of Kentucky, Lexington, Kentucky, United States of America; Pennsylvania State University Main Campus: The Pennsylvania State University - University Park Campus, UNITED STATES OF AMERICA

## Abstract

**Background:**

Pressure-sensing insoles offer a portable way to quantify human movement biomechanics beyond the lab, enabling real-world monitoring. A common objective with these insoles is to estimate ground reaction forces (GRFs). However, force estimates from insoles typically exhibit a magnitude bias—underestimating or overestimating GRFs—that can vary across individuals and contexts. Conventional force scaling approaches can correct for this bias, but they rely on ground truth force plate data for calibration. This is often impractical for remote or longitudinal data collection, particularly for individuals with lower-limb injuries or disabilities.

**Objective:**

The purpose of this study was to introduce and evaluate a new force scaling method for pressure insoles to enable more accurate estimation of vertical GRFs without laboratory calibration or access to ground truth force plate data, and which could be applied to individuals with lower-limb injuries or disabilities.

**Methods:**

The new method combines epoch-based and physics-based approaches to perform periodic and dynamic force scaling based on a user’s body weight, in a way that is well-suited for use in real-world patient monitoring, and which does not require an individual to weight-bear on a single limb. This force scaling method was evaluated across different participants and activities representative of daily living and rehabilitation exercises.

**Results:**

Compared to nominal (unscaled) insole force estimates, the new scaling method reduced GRF errors by an average of 54–72%, achieving average errors on the order of 0.11–0.16 body weights. The new method worked reliably for different individuals, both limbs, and a variety of tasks and exercises.

**Conclusions:**

This method provides a practical, generalizable, and effective approach for improving vertical GRF estimation from pressure insoles during remote, longitudinal monitoring without in-lab calibration or force plates. This could improve GRF assessment outside clinical or laboratory settings and enable clinicians, researchers, and users to more accurately monitor musculoskeletal loading and other biomechanical metrics during rehabilitation, recovery, and physical activities in real-world environments.

## Introduction

Pressure insoles provide a portable way to measure human movement biomechanics outside the clinic or research lab [1–4]. This capability is useful for a range of applications, including biomechanics research, clinical evaluation, rehabilitation, sports, remote monitoring, and longitudinal data collection [5–8]. A common goal is to obtain metrics that align with lab-based measures like ground reaction forces (GRFs) [9–11]; however, accurate biomechanical metrics are needed for reliable and valid insights when using pressure insoles for real-world monitoring.

GRFs are fundamental to understanding human movement, musculoskeletal loading, injury risk, and rehabilitation outcomes. However, measuring GRFs traditionally requires laboratory-grade equipment, such as a force plate or force-plate instrumented treadmill (e.g., [12–14]). GRFs are the forces exerted by the ground on the part of the person that is in contact with it (e.g., the outsole of a shoe). Force plates provide high-accuracy and comprehensive (e.g., 3D) measures of GRFs, but are expensive, immobile, and impractical for longitudinal studies and real-world settings. In contrast, pressure insoles are less-expensive, portable, and more practical to use in the real-world, but provide less accurate and less comprehensive force metrics. For instance, pressure insoles typically measure the 1D pressure (or 1D scalar force) between the shoe insole and the person’s foot. For these and other reasons, the GRF and pressure insole derived forces are similar and related, but not identical.

A key methodological challenge is determining how precisely to use pressure insole signals to estimate GRF. Total force estimates from pressure insoles are often highly correlated with the vertical component of GRFs (e.g., [15]), and this correlation is sufficient for certain applications. However, pressure insole force estimates tend to exhibit a bias in magnitude relative to the true GRFs. Some insoles systematically underestimate GRFs while others systematically overestimate GRFs. The magnitude of this bias varies across people, footwear, tasks, and other contexts and factors (e.g., [16]). This bias can be problematic for applications where absolute GRF accuracy is desirable. In these situations, pressure insole signal transformation (e.g., pressure calibration, force scaling) is typically needed to yield sufficiently accurate GRF estimates.

Scaling the total insole force is a common technique used to account for measurement bias in the research lab [17]. Force scaling is straightforward in short-term, controlled, laboratory studies, but complicated and limited in long-term, unconstrained, or remote settings. In the lab, force plate data can be measured synchronously, then used as a reference to identify scaling factors or train models to improve GRF estimation accuracy from pressure insoles. When ground truth training data are available, many different scaling approaches have been shown to be practical and effective (e.g., [12–14]), particularly for a constrained set of tasks (e.g., straight-ahead treadmill walking or running). Scaling methods range from simple to complex, and advances in machine learning have spurred the development of a wide range of more advanced force calibration and estimation methods (e.g., [18]).

However, estimating GRFs accurately and reliably in unconstrained, real-world (i.e., outside of the lab) environments is more challenging. There is little or no ground truth GRF reference data from which to scale insole force data or train calibration models, particularly for longitudinal assessments where individuals do not return to the lab for frequent re-calibration.

Consequently, existing scaling or calibration methods are generally not applicable, or have limitations with respect to their generalizability or prolonged use in unconstrained environments or with certain patient populations. Simple static calibrations (e.g., tabletop testing) exist, but often do not translate to accurate GRF estimates once an insole is placed inside a person’s shoe and used during real-world or dynamic activities [17,19]. Calibration methods that do not require force plates have also been developed that use standing on a single limb or level ground walking as training data to improve GRF estimates [20]. However, there are practical and user experience issues with these kinds of calibrations; for instance, if the user is recovering from a leg injury or has strength or stability issues, then balancing on one foot or walking without a physical aid to calibrate an insole to body weight (BW) is often not feasible.

The overarching problem is that there is a lack of methods to address the pressure insole force bias issue and to scale forces to accurately estimate GRFs during prolonged, remote, real-world monitoring for individuals with lower limb injuries or disabilities. We encountered this problem while planning a longitudinal remote data collection with pressure insoles involving patients after tibial shaft fracture surgery.

We sought accurate estimates of the vertical GRF from pressure insoles during daily life to use as an input to algorithms we previously developed to monitor tibial bone forces—forces which we envision could serve as useful biomarker for injury prevention or rehabilitation applications [19,21,22]. These algorithms use pressure insole-based forces, sometimes in combination with inertial measurement unit (IMU) data, to estimate targeted musculoskeletal loading or joint kinetics outcomes, without the need for comprehensive kinematics or kinetics measurements from lab instrumentation.

We needed a method that worked reliably for different patients (e.g., men and women of different heights, weights, and foot sizes), both limbs, multi-month monitoring, and a wide array of activities of daily living performed in unconstrained environments (e.g., indoor, outdoor, different terrains). We also needed a method that was not time-consuming or burdensome for patients with lower-limb injuries or disabilities, and ideally would be transparent in their daily life.

Compatibility with other patient and practical constraints were also critical. For instance, these patients are non-weightbearing after surgery, and often they do not return to the research lab (or clinic) before they begin gradually re-loading their limb based on guidance from their clinical care team. Therefore, it is impractical to perform a patient-specific lab calibration before these individuals begin wearing the pressure insoles for at-home monitoring.

We therefore set out to solve this problem of how to scale pressure insole forces to more accurately estimate vertical GRFs for long-term remote monitoring—without the need for recurring lab visits or training data from force plates. Here we introduce the novel method we developed, then evaluate its accuracy for estimating GRF during common locomotor and rehabilitation activities.

## Methods

### Force scaling method

We developed a force scaling method that could be applied to remote pressure insole data to improve vertical GRF estimation accuracy. This method does not require force plate training data or for users to perform any calibration activities. Below we briefly summarize our thinking behind the method, followed by details on how we implemented and evaluated it. Appendix A contains an extended discussion of our thought process, assumptions, and construct validity underlying the method. For brevity throughout this manuscript, references to GRF are referring to vertical GRF, unless otherwise stated.

The new method combines epoch-based and physics-based approaches to perform periodic and dynamic force scaling in a way that is well-suited and scalable for use in real-world settings. During daily life, a person performs a variety of static and dynamic activities. Some of these activities involve GRFs only transmitted through their feet (e.g., during standing, walking), while others involve GRFs that are partially transmitted through other body parts (e.g., during sitting, leaning). During unsupervised remote monitoring, we do not have a ground truth measure of GRFs, nor do we know precisely what someone is doing at a given moment. However, based on Newton’s 3^rd^ law we expect that, in general, the average GRF over a sufficient time period (which we call an epoch) should be approximately equal to the person’s BW. So, if we can identify or approximate epochs when the GRF is transmitted only through the person’s feet (and pressure insoles), then we can use these training epochs and the relationship between GRF and BW to compute force scaling factors.

Using this method, the force scaling factor can vary over time, and can be applied to both training and non-training epochs. In theory, this scaling method should bring pressure insole force magnitudes into closer absolute agreement with GRF magnitudes. This would improve the accuracy of GRF estimates from pressure insoles in a way that is compatible with and scalable for remote longitudinal monitoring in the real-world—without the need for training data, force plates, or additional sensors.

The schematic diagram in Fig 1 overviews the new force scaling method. After performing the initial data processing (Step 1) and defining epoch parameters (Step 2), the unscaled force data from the pressure insoles are split into epochs (time windows). Next, scaling factors are computed, for each training epoch (Step 3) and on average across all training epochs (Step 4). Then, force scaling is performed on each training and non-training epoch (Step 5). Finally, the epochs are concatenated to reassemble the full time-series of scaled forces (Step 6). The only two parameters that need to be defined are the epoch length and training threshold. This epoch-based approach can be thought of as a sliding window over a prolonged time-series of data (e.g., hours, days, weeks, months, years), where each window defines a distinct, non-overlapping time interval.

**Fig 1:**
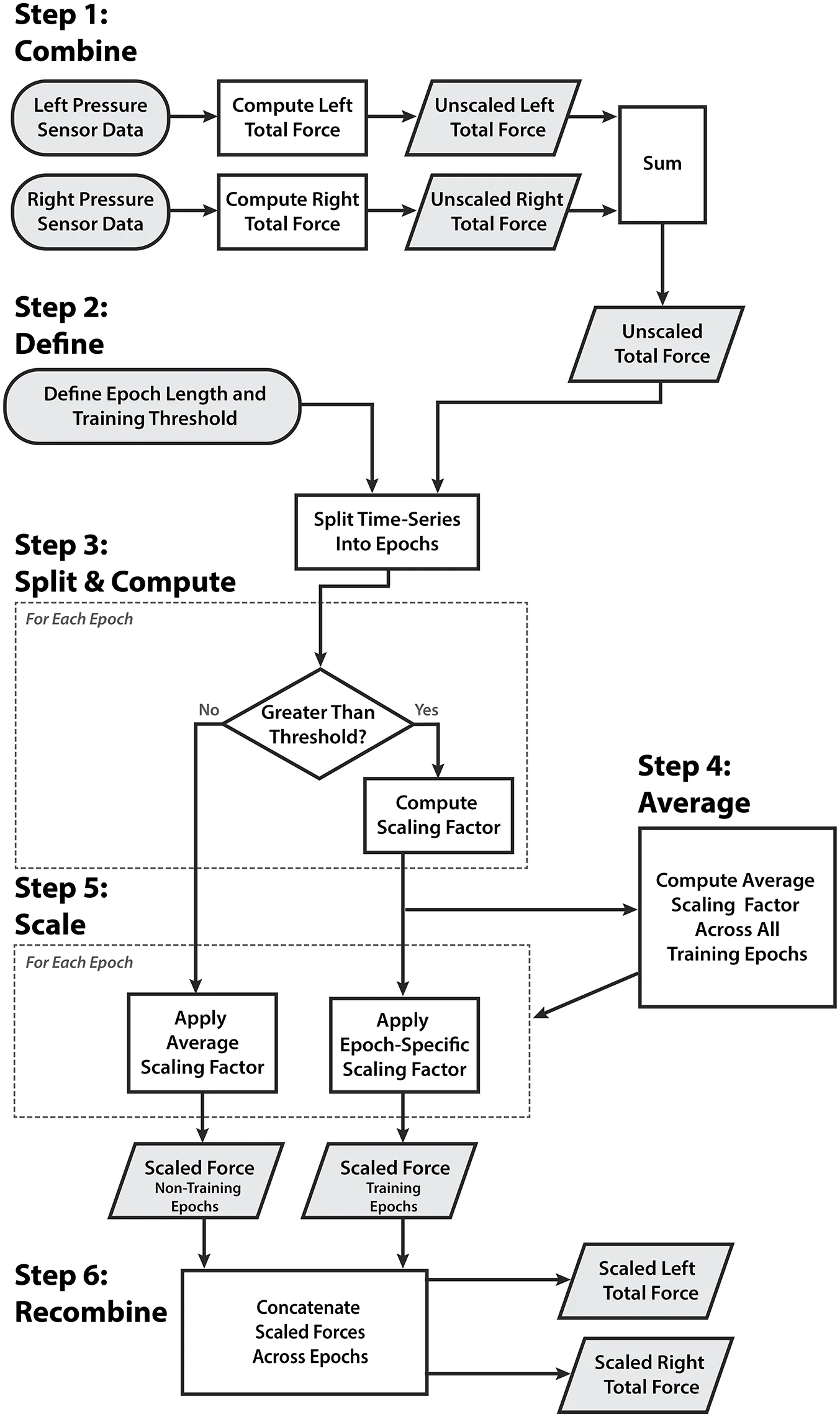
Schematic diagram overviewing the new force scaling method.

### Human subject experiment

We analyzed a human subject data set from a lab study to evaluate the accuracy of the unscaled and scaled pressure insole forces (Fig 1) relative to ground truth GRFs from in-lab force plates. The dataset was from 20 healthy individuals (10F/10M; age: 25.2 ± 4.6 years; height: 1.7 ± 0.1 m; mass: 68.5 ± 9.9 kg) who completed a series of common locomotor and rehabilitation exercises that spanned from sitting and standing to walking, running, calf raises, and jumping (see full list in Appendix B). This study was collected under a protocol approved by the institutional review board at the University of Kentucky, and individuals gave informed consent prior to participating. Participant recruitment spanned the period from 07/23/2024 through 11/20/2024.

We extracted data from the exercises that included complete and synchronous force plate and pressure insole data for each leg. Trials were excluded if both feet were on the same force plate, since this prevented ground truth GRF measurement for a single limb. In total, we analyzed about 25 exercises per participant. GRFs were collected at 2000 Hz using a force-instrumented split-belt treadmill (Bertec). For brevity, the term force plate is used in these methods to refer to each belt of the force-instrumented treadmill. The experimental methods and application of the force scaling method would be the same regardless of whether in-lab data were collected from a force-instrumented treadmill or in-ground force plates. Pressure insole data were collected at 100 Hz (OpenGo, Moticon ReGo AG, Munich, Germany), following a manufacturer-recommended initial warm-up period for sensor auto-zeroing. The insoles were OpenGo sensor insoles, which are instrumented with 16 plantar pressure sensors and a 6-axis inertial measurement unit. Data were collected and extracted using the OpenGo application (version 03.08.01.11882), using the continuous recording mode. Unscaled force refers to the total force output by the OpenGo software, based on summing across all pressure sensors (Fig 1). All participants wore New Balance WR662 shoes. Prior to analysis, all forces were normalized by participant BW, which was measured in a static calibration trial prior to the start of the experiment.

### Data synchronization

Synchronization between the pressure insoles and GRFs was performed in two stages. First, each exercise began with the participant tapping their right and then left heel on the ground (i.e., instrumented treadmill). These impact peaks in the force plate and pressure insole data were aligned. Second, we performed cross-correlation over the entire exercise duration to confirm or fine-tune the temporal synchronization between the force-instrumented treadmill and pressure data. During this process, the GRF data were resampled to match the 100 Hz sampling frequency of the insoles. To focus analysis on each exercise, the start and end of each trial was cropped to remove the foot tapping transient and any static standing or waiting period.

### Force scaling parameters

We defined epoch parameters and applied the scaling method (Fig 1) to the pressure insole data to compute the scaled force. We defined constant length epochs to be 5 seconds and the epoch training threshold to be 0.8 BWs. These parameters were based on pilot testing and the judgement of the research team. We also explored alternatives, and found that the force scaling method was relatively insensitive to other parameter values when applied to this dataset. For instance, the force scaling accuracy results were similar for epoch lengths from 3–30 seconds, and for thresholds from 0.5 to 1 BW in this study (Appendix E). For clarity, the in-lab force plate data were not used to scale the pressure insole forces or to compute scaling factors, only as ground truth values against which to benchmark accuracy (as detailed below).

### Analysis

The primary outcome metric was root mean square error (RMSE)—capturing how accurately the scaled and unscaled insole forces estimated the lab-based GRFs, quantified in terms of BW. We computed RMSE (i) for the full time-series waveform for each exercise, and (ii) for only the top 10% of GRF magnitudes. The top 10% of GRFs metric was computed because for applications related to tissue microdamage and stimulation, higher forces dominate. This is because microdamage and stimulation are exponentially related to tissue force. Thus, it was important to isolate and understand accuracy for higher GRFs. For instance, in our research on patients after tibial fixation surgery, we seek to understand how bone loading stimuli early in rehabilitation impact long-term recovery outcomes.

Secondarily, we also performed paired t-tests to determine if the scaled insole force errors were significantly lower than the unscaled insole force errors. A total of 4 statistical comparisons were performed across participants, including for right and left legs, and for each of the RMSE metrics (full and top 10%). We used a statistical significance level of 0.05 and applied a Holm-Sidak correction to account for multiple comparisons.

## Results

The force scaling method resulted in average RMSEs of 0.11 BW for the full GRF time-series and 0.16 BW for the top 10% of GRFs (Fig 2). Representative waveforms from one participant during a subset of tasks are depicted in Fig 3. RMSE results are broken down by task for the left leg (Tables 1 and 2). For brevity, tasks performed only on the right leg were omitted from Tables 1 and 2, since the left foot was not in contact with the ground and the RMSEs under the left leg were close to zero.

**Table 1:** RMSE in BW between Left Insole Force and GRF (full waveform) – By Task.

Task	Unscaled RMSE	Scaled RMSE
Double Limb Counter Movement Jump	0.27 ± 0.11	0.1 ± 0.02
Double Limb Calf Raise	0.41 ± 0.16	0.15 ± 0.04
Double Limb Jump to Left Single Limb Landing	0.36 ± 0.13	0.15 ± 0.03
Double Limb Jump to Right Single Limb Landing	0.22 ± 0.1	0.09 ± 0.02
Double Limb Stance	0.17 ± 0.11	0.03 ± 0.02
Double Vertical Jump	0.43 ± 0.14	0.2 ± 0.1
High Pogos	0.46 ± 0.15	0.14 ± 0.03
Low Pogos	0.5 ± 0.16	0.13 ± 0.06
Double Limb Rebounding Calf Raises	0.37 ± 0.14	0.14 ± 0.03
Left Single Limb Rebounding Calf Raises	0.61 ± 0.24	0.17 ± 0.05
Left Single Limb Calf Raises	0.58 ± 0.22	0.14 ± 0.05
Left Single Limb Pogos	0.66 ± 0.24	0.16 ± 0.03
Left Single Limb Seated Calf Raises	0.1 ± 0.06	0.07 ± 0.03
Left Single Limb Counter Movement Jump	0.31 ± 0.14	0.16 ± 0.02
Left Single Limb Stance	0.48 ± 0.29	0.04 ± 0.01
Squatting	0.18 ± 0.12	0.07 ± 0.03
Running at Self-Selected Speed	0.56 ± 0.17	0.13 ± 0.03
Walking at Self-Selected Speed	0.34 ± 0.1	0.1 ± 0.03
Walking at 1.3 m/s	0.35 ± 0.11	0.11 ± 0.03

**Table 2:** RMSE in BW between Left Insole Force and Top 10% of GRF – By Task.

Task	Unscaled RMSE	Scaled RMSE
Double Limb Counter Movement Jump	0.54 ± 0.22	0.2 ± 0.05
Double Limb Calf Raise	0.33 ± 0.16	0.12 ± 0.04
Double Limb Jump to Left Single Limb Landing	0.82 ± 0.29	0.36 ± 0.07
Double Limb Jump to Right Single Limb Landing	0.4 ± 0.19	0.15 ± 0.05
Double Limb Stance	0.17 ± 0.12	0.03 ± 0.02
Double Vertical Jump	0.77 ± 0.27	0.29 ± 0.12
High Pogos	1.04 ± 0.4	0.24 ± 0.08
Low Pogos	1.1 ± 0.38	0.21 ± 0.16
Double Limb Rebounding Calf Raises	0.42 ± 0.19	0.16 ± 0.04
Left Single Limb Rebounding Calf Raises	0.81 ± 0.35	0.18 ± 0.09
Left Single Limb Calf Raises	0.59 ± 0.23	0.11 ± 0.04
Left Single Limb Pogos	1.31 ± 0.51	0.22 ± 0.07
Left Single Limb Seated Calf Raises	0.12 ± 0.08	0.08 ± 0.04
Left Single Limb Counter Movement Jump	0.7 ± 0.28	0.36 ± 0.09
Left Single Limb Stance	0.48 ± 0.28	0.04 ± 0.02
Squatting	0.24 ± 0.17	0.1 ± 0.05
Running at Self-Selected Speed	1.28 ± 0.44	0.17 ± 0.04
Walking at Self-Selected Speed	0.54 ± 0.18	0.13 ± 0.07
Walking at 1.3 m/s	0.55 ± 0.2	0.14 ± 0.06

**Fig 2:**
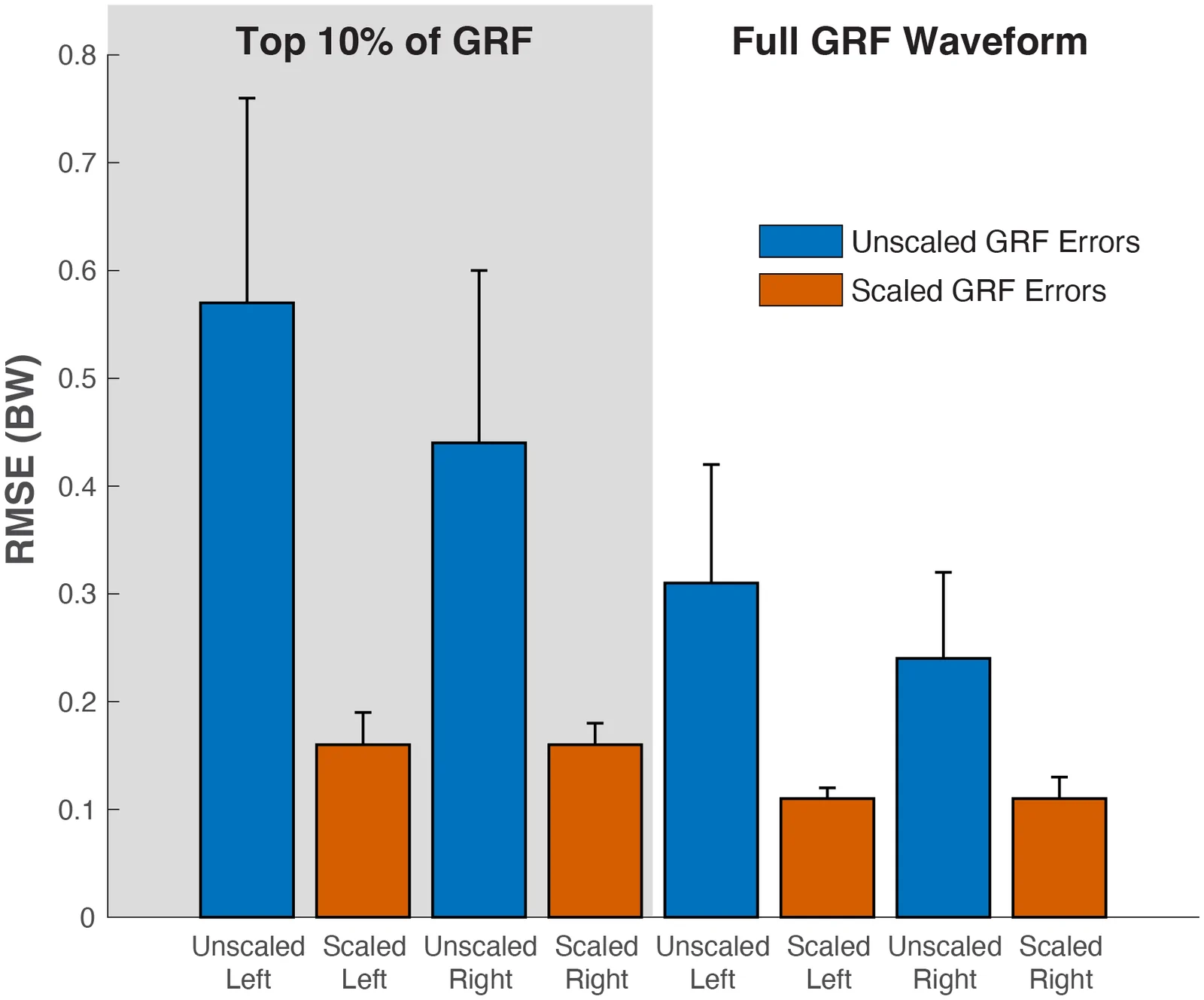
RMSE in units of BW between insole forces and GRF. The inter-subject averages and standard deviations across all exercises are depicted. RMSEs were significantly lower after the force scaling method was applied (p < 1e-6 for all unscaled vs. scaled comparisons). See Tables C2–C5 in S1 Appendix for the subject-specific results from which these summary metrics were computed.

**Fig 3:**
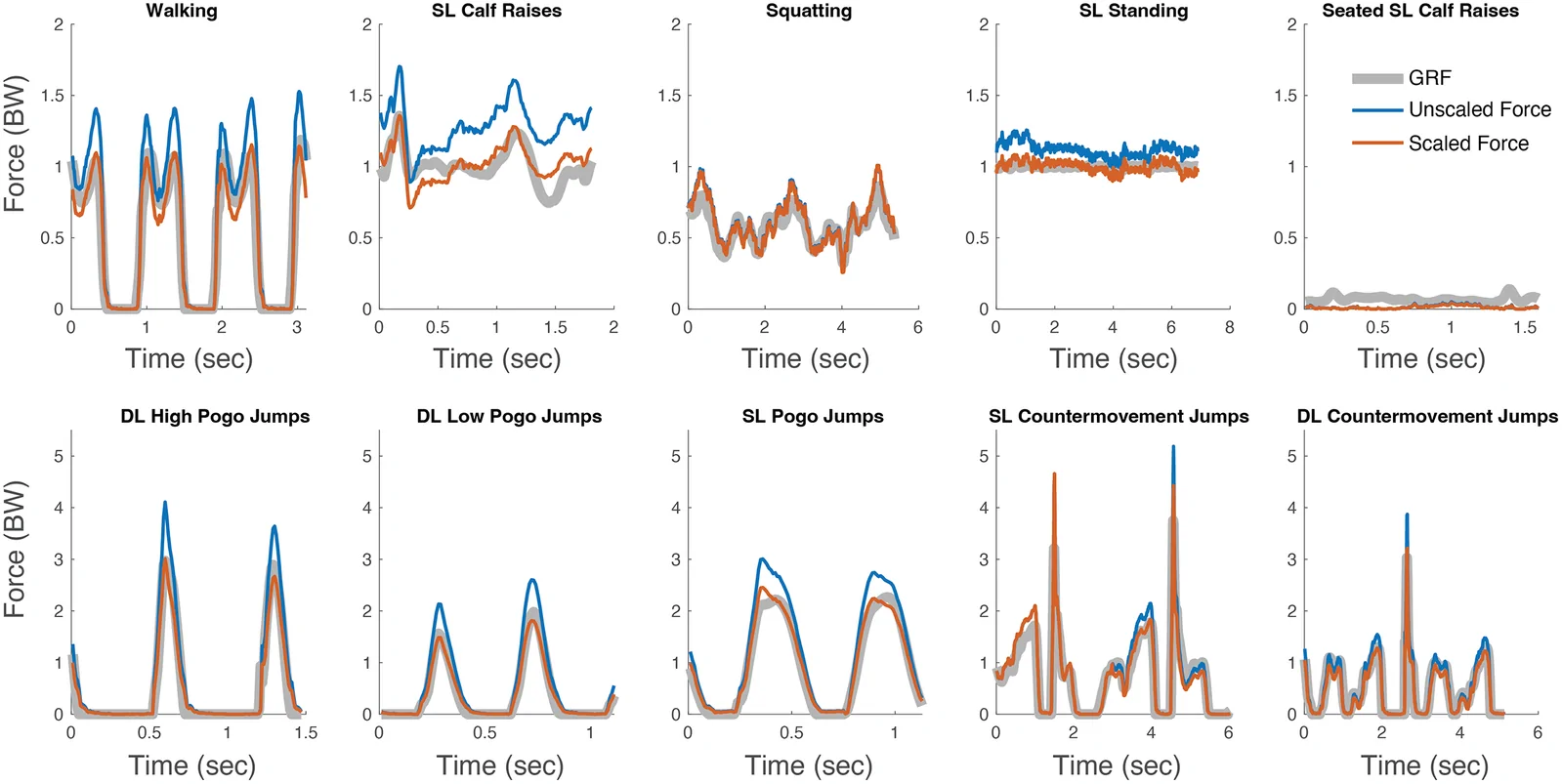
Representative subject data during a subset of tasks. The ground truth GRFs (from force plates, gray thick line) are better approximated by the scaled forces (red line) than by the unscaled forces (blue line) from the pressure insoles. SL = single limb; DL = double limb.

Results were similar for right and left legs for all RMSE metrics after force scaling.

Extended results are included in Appendix C, including RMSE average results (Table C1 in S1 Appendix) and RMSEs broken down by participant (Tables C2–C5 in S1 Appendix) and by task for the right leg (Tables C6 and C7 in S1 Appendix). For brevity, tasks performed only on the left leg were omitted from Tables C6 and C7 in S1 Appendix, since the right foot was not in contact with the ground and the RMSEs under the right leg were close to zero.

RMSEs were significantly lower after the force scaling method was applied (p < 1e-6 for all comparisons; Fig 2). Across the full time-series, the force scaling method reduced RMSE by 54−65%, from an average error of 0.24–0.31 BW unscaled to 0.11 BW scaled. For the top 10% of GRFs, the force scaling method reduced RMSE by 54−72%, from an average error of 0.44–0.57 BW unscaled to 0.16 BW scaled.

## Discussion

We developed a force scaling method for pressure insoles that is practical to apply during remote, longitudinal data collections and we evaluated its accuracy against ground truth GRF measures. This new method reduced GRF bias and estimation errors—by 54–72% in this study—relative to estimating GRFs directly from pressure insole forces. The method worked for different individuals (including men and women of different height, weight, and foot size) and both limbs (Fig 2 and Tables C2–C5 in S1 Appendix), and a variety of exercises (Tables 1, 2, C6 and C7 in S1 Appendix), which are representative of common activities of daily life (e.g., standing, sitting, walking, running, jumping, squatting, calf raises).

### Accuracy relative to prior work

This new force scaling method achieved GRF estimation accuracy that was reasonable and in line with previously published methods. The level of accuracy that is needed will depend on the application; however, qualitatively, our view is that being able to estimate GRFs with average errors on the order of 0.11 BW and peak (top 10% of GRF) errors of 0.16 BW is likely to be useful for various research, clinical, and consumer applications. See extended discussion on this topic in the ‘Other Considerations and Expectations’ section below.

Quantitatively, the observed RMSE values align with prior research on other pressure insole force scaling methods. For instance, Choi et al. [12], Hajizadeh et al. [13], and Olugbon et al. [14] detailed different trained models for estimating GRF during tasks like walking, running, squatting, and sit-to-stand, and each reported average RMSEs between 0.04–0.16 BW for different tasks. This accuracy range aligns well with our participant- and task-specific RMSE findings in Tables C2–C7 in S1 Appendix.

For tasks with larger peak forces (e.g., running, jumping, landing), RMSEs tend to be higher, and may be more variable. For instance, Carter et al. [23] reported participant-specific RMSE ranging from 0.09 to 0.62 BW after developing a model to estimate GRFs from pressure insoles during different running conditions. In our current study, RMSEs were generally higher for tasks like jumping and landing than sitting and standing; however, even the most dynamic and high-impact jumping exercises in this study still only exhibited relatively small or modest errors after force scaling (Tables 1, 2, C6 and C7 in S1 Appendix). In fact, the higher impact activities exhibited the greatest reductions in RMSE after the force scaling method was applied, which is promising for the generalizability of this method across tasks and task intensities.

### Generalizability of method

The new force scaling method appeared to work similarly well across all participants, including men and women, and people of different size and weight. Across participants, average RMSE only varied from 0.09 to 0.14 BW on the left side, and from 0.08 to 0.13 BW on the right side. In terms of the top 10% of GRFs, the average RMSE across participants ranged from 0.13 to 0.23 on the left side and from 0.12 to 0.2 on the right side.

Of note, this study was only performed with one brand of pressure insole, though we believe this force scaling method should be equally applicable to other pressure insole systems based on the construct and rationale detailed in Appendix A.

In theory, the scaling factors might vary between right and left insoles. However, the results of this study showed similar RMSE for right and left legs, suggesting this was not a significant confound, on average. Most importantly, both left and right side RMSE improved when applying the force scaling methods.

In practice, this force scaling method is intended to supplement and enhance—not replace—any factory calibrations or in situ calibrations that are recommended by pressure insole manufacturers prior to data collection.

### Practicality of method

Our work sought to extend the practicality and utility of force scaling methods for unsupervised, remote, or longitudinal monitoring in unconstrained, real-world (e.g., at home) settings. In particular, for our ongoing clinical study, we specifically sought methods that could be used to monitor the recovery of patients who have had a surgical fixation of their tibia. The key attributes of this force scaling method that make it practical are:

- It does not require GRF (ground truth) reference data
- It does not require a person to be able to balance or weight-bear on a single limb
- It does not require users to return to the lab or clinic for calibration or recalibration
- It does not require user effort or action (i.e., it is not time-consuming or burdensome)
- It is performed continuously (on a rolling basis) in the background during daily life
- It does not rely on activity recognition or any other sensors

### Limitations of method

There are a few limitations of this force scaling method to acknowledge. Some are general limitations, while others are limitations to certain variations of this method, and yet others are limitations, in theory, but expected to be minor in practice.

Generally, this method may be less applicable or accurate for tasks with large shear GRFs (anterior-posterior or mediolateral). We do not expect this to be problematic for our study population because patients recovering from tibial fracture surgery have limited ambulation and are not expected to perform activities with large shear GRFs (e.g., sharp cutting maneuvers while running). However, this method may have limitations for certain high intensity use cases. It may need to be evaluated—and potentially tuned or refined—for certain non-rehabilitation or sport-specific uses like cutting or pivoting.

The specific implementation of the method detailed in this manuscript computed a single scaling factor per foot per epoch, which may not fully correct for potential differences due to sensor density and distribution across an insole. In practice, this method could be extended to compute more than one force scaling factor per foot. For instance, one force scaling factor could be computed for timepoints when the center of pressure is on the back (posterior) half of the user’s foot, while another might be computed for timepoints when the center of pressure is on the front (anterior) half of the user’s foot. This and other variations of the force scaling factors could be explored in future work, or used to tailor the method for specific use cases.

In theory, substantial changes in body weight or apparent weight could influence this scaling method. However, these effects are unlikely to meaningfully limit most applications, particularly in clinical contexts. True weight changes may occur with significant weight gain or loss, while apparent weight changes can arise from carrying external loads or variations in clothing.

In practice, the impact on the force scaling method is expected to be minimal. Sustained load carriage typically only occurs for a small fraction of the day, and clothing weight differences are minor compared to body weight. For example, lifting a load equal to 20% of body weight for 5 seconds within a 60-second training epoch would introduce less than 2% error in GRF estimates using this method. Clothing-related variation is similarly limited: seasonal differences of approximately 1–3 kg correspond to about 1–4% of average adult body weight in the U.S., producing errors of comparable magnitude.

Exceptions do exist. Individuals who regularly carry substantial loads (e.g., a parent holding an infant) may require additional consideration, and the method should not be applied without accounting for these conditions. Nonetheless, such scenarios are not expected in our target population, as post-operative tibial fracture patients are unlikely to engage in routine or prolonged heavy load carriage.

Given these considerations, we implemented a simple “greater than X” thresholding approach in this study. Alternatively, this epoch training threshold could be defined other ways, such as with a “greater than X and less than Y” range or using more sophisticated techniques for identifying training epochs. In practice, the epoch training criteria could be tailored for specific use cases. Also, for true long-term monitoring there may be other simple practices, such as a weekly or monthly weigh-in, which patients perform on a bathroom scale and which are used in the longitudinal trend analysis or other biomechanical analyses to improve accuracy.

### Other considerations and expectations

We expect this force scaling method is compatible with different environments (e.g., indoor, outdoor) and terrains (e.g., hard or soft ground, slopes). For rigorous evaluation of accuracy we needed to collect ground truth GRF data in the lab, which limited the breadth of tasks and terrains we evaluated. However, the force scaling method is based on Newton’s 3^rd^ law (Appendix A), which is agnostic to the environment, individual, task, or terrain.

We also expect this force scaling method is applicable across different types of footwear and pressure insole products; however, this was not explicitly tested in this protocol. We are not aware of, nor have we been able to conceive of, any confounds that would prevent using this force scaling method for remote or longitudinal data collections with other pressure insoles.

We consider this method to be reasonable (logically) and well-justified (scientifically), and expect that it should be broadly applicable and scalable for remote and longitudinal data collections. Though, admittedly, this is difficult to prove empirically since ground truth GRF data is not available in the real-world. Nonetheless, the construct validity (Appendix A) and initial validation (Fig 2 and Tables 1, 2 and C2–C7 in S1 Appendix) support using the proposed method. If nothing else, the findings here indicate that this force scaling method is better than doing nothing (i.e., using unscaled forces), given that GRF estimation accuracy improved across every RMSE metric, individual, leg, and task.

We expect that the GRF accuracy observed after applying the force scaling method is sufficient for certain use cases, but not all use cases. The accuracy results are promising and likely adequate for some remote therapeutic monitoring or telerehabilitation applications. Peak GRFs during most of the rehabilitation exercises in this study ranged from approximately 1–4 BW. The average and peak GRF errors of 0.11 and 0.16 BW (Fig 2), respectively, are comparatively small.

It is also helpful to contextualize these results relative to the current standard of care. Post-operative patients are typically sent home without any ability for the medical team or the patient to monitor limb loading. In the weeks following surgery, patients are often instructed to place 0.25 BW (i.e., 25% weightbearing) on their injured limb, and then gradually increase this to 0.5, 0.75, and 1 BW. Achieving GRF estimation errors that are substantially smaller than these 0.25 BW increments further suggests that the accuracy is likely sufficient for certain clinical uses. For additional perspective on the question of “what accuracy is good enough?”, including quantitative insights from prior literature and real-world healthcare and fitness devices, see Elstub et al. [19].

There are multiple different variations of this force scaling method, but precise implementation will depend on the use case or study goals. Variations include different ways of defining model input parameters or output timescales. See Appendix F for examples and additional details.

## Conclusions

We introduced a new force scaling method for pressure insoles and demonstrated its ability to enhance vertical GRF estimates, without requiring laboratory calibration or access to ground truth force plate data. This method shows promise as a practical and generalizable way to accurately estimate GRFs from pressure insoles during remote, unconstrained longitudinal data collections, particularly for rehabilitation or clinical monitoring purposes. As such, this method has the potential to help democratize access to high-fidelity GRF estimation—enabling clinicians, researchers, and everyday users to more accurately quantify biomechanical loading in the real world.

## Supporting information

S1 Appendix Appendices A-F. (DOCX)
