# Supplementary material for: A novel force scaling method for ground reaction force estimation from pressure insoles to enable long-term remote monitoring
Source: PLoS One. 2026 Oct 26;21(10):e0358957. doi: 10.1371/journal.pone.0358957 (PMC13704854; doi:10.1371/journal.pone.0358957)
Supplement: Supplementary file 1 [file pone.0358957.s001.docx]

**Appendix A: Force scaling method - thought process and construct validity**

In the research lab, force plates provide the ground truth GRF. In the real-world, we needed an analogous ground truth, or at least a reasonable proxy, that could be used to scale pressure insole forces and mitigate measurement bias. We identified body weight (BW) as the most reliable and useful real-world proxy, then developed a method for leveraging the relationship between BW and GRF to dynamically and adaptively scale insole forces to improve the accuracy of GRF estimation. To understand the construct validity of this approach, it is easiest to explain our thought-process, starting with static posture and then expanding into dynamic movements and real-world applications.

Let’s start with the simplest case: Imagine someone is standing statically on one or both of their feet. Regardless of whether they are standing on their heels, toes, or flat-footed, the GRF equals one BW. This is true at every instant in time. Even if the standing was quasi-static. For instance, the person was fidgeting or slowly shifting their BW, we would still expect this relationship (GRF ≈ 1 BW) to be a reasonable approximation. As such, we could divide 1 BW by the pressure insole force at every instant in time to compute a force scaling factor. We could then multiply the pressure insole force by the scaling factor to estimate the GRF with higher accuracy (relative to the unscaled pressure insole force).

But for dynamic movements this relationship no longer holds true. GRFs fluctuate above and below one BW during motion. However, over a sufficient period of time (which we call an *epoch*), the average GRF is expected to equal about 1 BW. This relationship is definitively true if someone is not changing their elevation; for instance, if someone is walking on level ground, or running, hopping, skipping or any other movement. This relationship (average GRF over an epoch ≈ 1 BW) is also a reasonable approximation if someone is slightly or occasionally changing elevation. As such, we could divide 1 BW by the average pressure insole force during an epoch to compute a force scaling factor for that epoch. Again, we could multiply the unscaled pressure insole force by the scaling factor, and we would expect the resultant to provide a more accurate estimate of the GRF.

But for real-world activities, this relationship no longer holds true, at least not for every epoch. Until now, we have also assumed that all the GRF is going through the feet of the user. This is what allowed the direct force scaling between the pressure insoles and GRF (applied to the feet). However, in real-life people will sit or lie down (e.g., a portion of the GRF is transmitted via a chair through the buttocks), or lean on an object (e.g., a portion of the GRF is transmitted via the object through the hands or arms). In these exemplary cases, there will be epochs of time when the GRF applied to the feet is, on average, less than 1 BW.

This thought process led us to the following approach. We needed to be able to identify appropriate training epochs, which could be used to compute force scaling factors based on the expectation that the average GRF should approximately equal 1 BW. We needed to avoid computing force scaling factors during epochs that violate our assumptions (e.g., large elevation changes, or significant GRFs transmitted to parts of the body other than the plantar surface of the feet). We refer to these periods as non-training epochs. The pressure insole data during these non-training epochs still needs to be scaled, but using a scaling factor computed during one or more of the training epochs. The main text provides further implementation details and methodological variations related to how to define epoch length, how to identify training epochs, and how to perform scaling during non-training epochs.

**Appendix B: Exercises**

List of exercises:

- standing double limb
- standing on single limb (right and left)
- standing double limb calf raises
- standing single limb calf raises (right and left)
- seated single limb calf raises (right and left)
- body weight squats
- double limb low pogo jumps
- single limb low pogo jumps (right and left)
- double limb high pogo jumps
- single limb high pogo jumps (right and left)
- drop vertical jumps
- double limb counter movement jumps
- single limb counter movement jumps (right and left)
- double limb rebounding calf raises
- single limb rebounding calf raises (right and left)
- double limb jump to single limb landing (right and left)
- walking at 1.3 m/s
- walking at self-selected speed
- jogging at self-selected speed

For self-selected tasks, participants were instructed to choose a comfortable walking and jogging speed, respectively. For walking and running tasks, one minute of data were collected. For standing tasks, 30 seconds of data were collected. For other cyclic tasks, participants performed 10 repetitions per exercise.

**Appendix C: Subject- and task-specific RMSE results**

**Table C1.** RMSE in BW between Insole Forces and GRF. Inter-subject averages and standard deviations are reported. These results are visualized in bar plots in Fig 2.

|  | **Left Foot** | | **Right Foot** | |
| --- | --- | --- | --- | --- |
|  | **Unscaled** | **Scaled** | **Unscaled** | **Scaled** |
| **RMSE  (Full Waveform)** | 0.31 ± 0.11 | 0.11 ± 0.01 | 0.24 ± 0.08 | 0.11 ± 0.02 |
| **RMSE (Top 10% of GRFs)** | 0.57 ± 0.19 | 0.16 ± 0.03 | 0.44 ± 0.16 | 0.16 ± 0.02 |

**Table C2.** RMSE in BW between Left Insole Force and GRF (full waveform) - By Participant

**Table C3.** RMSE in BW between Right Insole Force and GRF (full waveform) - By Participant

**Table C4.** RMSE in BW between Left Insole Force and Top 10% of GRF – By Participant

**Table C5.** RMSE in BW between Right Insole Force and Top 10% of GRF - By Participant

**Table C6.** RMSE in BW between Right Insole Force and GRF (full waveform) - By Task

**Table C7.** RMSE in BW between Right Insole Force and Top 10% of GRF - By Task

**Appendix E: Sensitivity analysis on epoch length and training threshold**

For the primary analysis, we defined a nominal epoch length of 5 seconds and a nominal epoch training threshold of 0.8 BWs (see Methods). In addition, we performed a preliminary analysis to explore how sensitive the error results were to different epoch parameters. We found that the force scaling method was relatively insensitive to other parameter values within a reasonable range. For instance, the force scaling accuracy results were similar for epoch lengths from 3-30 seconds, and for thresholds from 0.5 to 1 BW in this study. For brevity, Tables E1 and E2 summarize RMSE results for the left foot only.

**Table E1.** RMSE in BW between Left Insole Force and GRF (full waveform) for different combinations of epoch length (in seconds) and training threshold (in BW). Inter-subject averages and standard deviations are reported.

|  | **0.5 BW** | **0.8 BW** | **1 BW** |
| --- | --- | --- | --- |
| **3 seconds** | 0.11 ± 0.01 | 0.11 ± 0.01 | 0.13 ± 0.02 |
| **5 seconds** | 0.11 ± 0.01 | 0.11 ± 0.01 | 0.12 ± 0.02 |
| **30 seconds** | 0.13 ± 0.02 | 0.13 ± 0.02 | 0.12 ± 0.02 |

**Table E2.** RMSE in BW between Insole Forces and Top 10% of GRF for different combinations of epoch length (in seconds) and training threshold (in BW). Inter-subject averages and standard deviations are reported.

|  | **0.5 BW** | **0.8 BW** | **1 BW** |
| --- | --- | --- | --- |
| **3 seconds** | 0.17 ± 0.02 | 0.17 ± 0.02 | 0.19 ± 0.05 |
| **5 seconds** | 0.16 ± 0.03 | 0.16 ± 0.03 | 0.18 ± 0.04 |
| **30 seconds** | 0.18 ± 0.05 | 0.18 ± 0.05 | 0.17 ± 0.03 |

**Appendix F: Variations of the Force Scaling Method**

There are various ways to define the two input parameters (epoch length and training threshold) in this force scaling method. Here we defined these parameters manually, but they could potentially be automated in the future. Epoch length might, for instance, be defined based on the nature of the data or tasks, by leveraging subject-matter expertise, or by automated data-driven approaches (e.g., based on the historical distribution of force data).

During in-lab evaluation trials (e.g., treadmill walking), we have pristine training epochs because all GRFs were transmitted through the participants’ feet, and people were not sitting or leaning on anything during the trial. As such, the epoch length could be shorter (e.g., 3-5 seconds). In real-world remote data collections, where there is more variability and uncertainty about a person’s activities and how they are physically interacting with the environment, it may be preferable to define longer epochs (e.g., 10-60 seconds).

Likewise, there are various ways to define the epoch training threshold to identify appropriate periods. Here we used a simple thresholding method. If the average insole force during an epoch was greater than a specified threshold (in units of BW), then the epoch was used as a training epoch. Alternatively, machine learning or other models could be used to dynamically adjust and/or define the epoch training threshold (or epoch length) based on an individual’s activity level or the distribution of insole forces recorded. Either of these parameters could be static, or these could be time-varying. Alternatively, training epochs may be identified through other means, such as by leveraging data from additional sensors (e.g., IMUs, fitness trackers, cameras) or user input (e.g., written descriptions, audio recordings, or activity tags).

This force scaling method could be applied in post-processing, or used in near-real-time or real-time applications. For our tibial fracture study, we intend to perform this epoch-based analysis offline during post-processing (e.g., performed at the end of each day). However, this method could also be implemented on-board the pressure insole to run in near-real-time; for instance, by scaling forces after each epoch (e.g., every 10-60 seconds). Alternatively, after sufficient data (e.g., several minutes) are collected to compute an average force scaling factor, then this method could be applied in real-time—by treating each instantaneous new sample of pressure data as a non-training epoch and applying an average or recently-computed force scaling factor to compute GRF. This is one example of a real-time implementation of this force scaling method. We expect that this variation would provide a reasonable real-time estimate of GRF, though accuracy may be further improved in post-processing when applying the epoch-based method to data over a longer timeframe (Fig 1).

GRFs are often not the final outcome metric, rather they are an intermediate metric, which is then used as an input to other models or algorithms. For instance, GRF data may be combined with motion data (e.g., from inertial measurement units) to estimate inverse dynamics or other musculoskeletal kinetics, or might be combined with reference data or a clinical look-up table to track rehabilitation progress. As such, this force scaling method (and variations thereof) may be used in conjunction with other data or biomechanical analyses, or with monitoring, biofeedback, or control systems that rely on GRFs.
